# Supplementary material for: End Sequence Analysis Toolkit (ESAT) expands the extractable information from single-cell RNA-seq data
Source: Genome Res. 2016 Oct;26(10):1397–410. doi: 10.1101/gr.207902.116 (PMC5052061; doi:10.1101/gr.207902.116)
Supplement: Supplemental Material [file supp_gr.207902.116_Supp_Materials.tar.gz › ESAT-esat_v0.1/doc/html/classes.html]

ESAT: Class Index


ESAT


- Main Page
- Related Pages
- Packages
- Classes
- Files

- Class List
- Class Index
- Class Hierarchy
- Class Members


All Classes Namespaces Files Functions Variables Enumerator Pages

Class Index

A | B | C | D | E | F | G | H | I | J | K | L | M | N | O | P | Q | R | S | T | U | W

|  |  |  |  |  |
| --- | --- | --- | --- | --- |
| A | ComputeFDR (broad.core.math) | G | MultipleAlignmentIO (broad.core.multiplealignment) | SAMFragmentWriter (broad.pda.samtools) |
|  | ComputeHypergeomtricEnrichment (broad.pda.geneexpression) |  | MultipleAlignmentIOFactory (broad.core.multiplealignment) | SAMPairedEndFileReader (broad.pda.samtools) |
| AbsolutePearsonFunction (broad.pda.geneexpression.clustering) | ComputeKmersForRegion (broad.core.siphy.tools.conservation) | GCContent (broad.pda.seq.rap) | MultiSampleScanPeakCaller (broad.pda.seq.protection) | SampleData (broad.pda.seq.protection) |
| AbstractAnnotation (umms.core.annotation) | ComputeMaxPercentileOfElements (broad.core.siphy.tools.conservation) | Gene (umms.core.annotation) | MultiScore (umms.core.model.score) | SampleData (broad.pda.seq.clip) |
| AbstractAnnotationCollection< T extends Annotation > (umms.core.annotation) | ComputeMIRScore (broad.core.primer3) | GeneCounts (broad.pda.seq.segmentation) | N | ScaledWindowParserListener (broad.core.siphy) |
| AbstractFastaParser (broad.core.sequence) | ComputeOriginalScore (broad.core.primer3) | GenericAlignmentDataModel (broad.pda.seq.segmentation) |  | ScanStatistics (broad.core.math) |
| AbstractListScore (umms.core.model.score) | ComputePercentageOfKmers (broad.core.siphy.tools.conservation) | GenericRootedTree< T > (broad.core.datastructures) | NeedlemanWunsch (jaligner) | ScanStatisticScore (umms.core.model.score) |
| AbstractPairedEndAlignment (umms.core.alignment) | ConnectDisconnectedTranscripts (umms.core.scripture.statistics) | GeneScore (broad.pda.seq.segmentation) | NeedlemanWunschGotoh (jaligner) | ScoreDiffGenes (broad.pda.geneexpression) |
| WindowProcessor< S extends WindowScore >.AbstractProcessor< S extends WindowScore > (umms.core.model.score) | ConservationUtils (broad.core.siphy) | GeneSetEnrichment (broad.projection.gsa) | NeighborAnalysis (broad.pda.gene) | ScoreMotifInstances (broad.core.motif.misc) |
| WindowScore.AbstractWindowScore (umms.core.model.score) | ConservedPWMScanner (broad.core.siphy) | GeneSetIntersect (broad.core.overlaputils) | NeighborsNullModel (broad.pda.gene) | SearchException (broad.core.motif) |
| AgilentArrayStats (broad.pda.geneexpression.agilent) | ContinuousDataAlignmentModel (broad.pda.seq.segmentation) | GeneTools (broad.pda.gene) | NewRatioScore (umms.core.model.score) | Sequence (broad.core.sequence) |
| AgilentProbe (broad.pda.geneexpression.agilent) | ConvertToDiscreteMatrix (broad.pda.differentialExpression) | TranscriptomeSpace.GeneTree (umms.core.coordinatesystem) | AlignmentModel.NodeIterator (umms.core.model) | Sequence (jaligner) |
| AgilentToGCT (broad.pda.geneexpression.agilent) | CoordinateSpace (umms.core.coordinatesystem) | GeneWindow (umms.core.feature) | JCSAlignmentModel.NodeIterator (umms.core.model) | SequenceMotif (broad.core.motif) |
| AgilentUtils (broad.pda.geneexpression.agilent) | CorrelationAgilentNanostring (broad.pda.geneexpression.agilent) | GeneWithIsoforms (broad.pda.gene) | O | SequenceMotifCluster (broad.core.motif) |
| Alignment (jaligner) | CorrelationBetweenReplicates (broad.pda.geneexpression.agilent) | GenomeCommandLineProgram (broad.pda.seq.rap) |  | SequenceParser (jaligner.util) |
| Alignment (umms.core.alignment) | CorrelationMatrix (broad.pda.geneexpression.clustering) | GenomeScoringProgram (broad.pda.seq.rap) | OrientedChromosomeTranscriptGraph (umms.core.scripture) | SequenceParserException (jaligner.util) |
| AlignmentModel.AlignmentCount (umms.core.model) | CountNums (broad.pda.geneexpression) | GenomeSpaceSampleData (broad.pda.seq.clip) | OverlapFilter (umms.core.annotation.filter) | SequenceRegion (broad.core.sequence) |
| JCSAlignmentModel.AlignmentCount (umms.core.model) | CountProbes (broad.pda.seq.rap) | GenomeSpaceSampleData (broad.pda.seq.protection) | IntervalTree< V >.OverlapIterator (broad.core.datastructures) | SequenceRegionWindow (broad.core.sequence) |
| AlignmentDataModel (broad.pda.seq.segmentation) | CountReads (broad.pda.seq.rap) | GenomeWindow (umms.core.feature) | P | SequenceUtils (broad.core.sequence) |
| AlignmentDataModelStats (broad.pda.seq.segmentation) | CountReads (broad.pda.seq.rap.rna) | GenomeWithGaps (broad.pda.seq.segmentation) |  | ShortBED (broad.core.annotation) |
| AlignmentExtractor (broad.core.multiplealignment) | CountScore (umms.core.model.score) | GenomeWithGaps2 (broad.pda.seq.segmentation) | PaGEJava (broad.pda.geneexpression) | ShortBEDReader (broad.core.annotation) |
| AlignmentModel (umms.core.model) | CoverageScore (umms.core.model.score) | GenomicAnnotation (broad.core.annotation) | Pair (jaligner.formats) | ShuffleBAM (broad.pda.seq.rap) |
| AlignmentPair (umms.core.alignment) | CrossJunctionPCRDesigner (broad.core.primer3) | GenomicAnnotationFilter< T extends LightweightGenomicAnnotation > (broad.core.annotation) | Pair< T1 > (broad.core.datastructures) | ShuffleBED (broad.pda.seq.rap) |
| Alignments (broad.pda.datastructures) | D | GenomicSpace (umms.core.coordinatesystem) | PairedAndProperFilter (nextgen.core.readFilters) | ShuffledIterator< T extends Annotation > (umms.core.coordinatesystem) |
| AlignmentsReader (broad.pda.datastructures) |  | GenomicSpanFilter (nextgen.core.readFilters) | PairedEndAlignmentFactory (umms.core.alignment) | SingleEndAlignment (umms.core.alignment) |
| AlignmentUtils (broad.pda.seq.alignment) | DefineGeneSignatures (broad.projection.gsa) | GFF (broad.core.annotation) | PairedEndFilter (umms.core.readFilters) | SingleInterval (umms.core.annotation) |
| AMEResultReader (broad.core.motif.meme) | DefineSourceAndSinkFromPairedEnds (broad.pda.seq.segmentation) | GFFReader (broad.core.annotation) | PairedEndReader (umms.core.readers) | SingularValueDecomposition (Jama) |
| Annotation (umms.core.annotation) | DifferentialExpression (broad.pda.differentialExpression) | GlobalSpliceFilter (broad.pda.seq.segmentation) | PairedEndReadIterator (broad.pda.samtools) | SkellamKLPeakCaller (broad.pda.seq.rap) |
| AnnotationCollection< T extends Annotation > (umms.core.annotation) | DifferentialScoring (broad.pda.differentialExpression) | GlobalStats (broad.pda.geneexpression) | PairedEndTDF (broad.pda.samtools) | SkellamScore (broad.pda.seq.rap) |
| AnnotationCounts (broad.pda.countreads) | Directions (jaligner) | GMTParser (broad.core.util) | PairedEndWriter (umms.core.writers) | SlideAndCalculate (broad.pda.seq.rap) |
| AnnotationFactory< T extends LightweightGenomicAnnotation > (broad.core.annotation) | DirectoryInstalledGenomeAssembly (broad.pda.feature.genome) | GrabProbes (broad.pda.differentialExpression) | PairedReadAlignment (umms.core.alignment) | SlideAndCount (broad.pda.seq.rap) |
| AnnotationFactoryFactory (broad.core.annotation) | DistanceToBED (broad.pda.seq.rap) | GrabSpecificColumns (broad.pda.geneexpression) | ParseException (broad.core.error) | SmatchLike (umms.core.alignment) |
| AnnotationFileReader (umms.core.annotation) | Distribution (broad.core.math) | GSAProjection (broad.pda.differentialExpression) | ParseGCTFile (broad.core.util) | SmithWatermanAlignment (umms.core.alignment) |
| AnnotationHandler (broad.core.annotation) | E | GSAScoring (broad.projection.gsa) | Path (broad.pda.seq.graph) | SmithWatermanGotoh (jaligner) |
| AnnotationList< T extends Annotation > (umms.core.annotation) |  | GsaUtils (broad.projection.gsa) | PCRDuplicateFilter (umms.core.readFilters) | SplicedReadFilter (umms.core.readFilters) |
| AnnotationOutOfBoundsException (umms.core.coordinatesystem) | ChromosomeWithBubblesJGraphT.EdgeSourceType (broad.pda.seq.graph) | GTFFileParser (broad.pda.annotation) | PeakFilter (broad.pda.seq.chip) | SplitAndTrimIndex (broad.pda.seq.fastq) |
| AnnotationReader< T extends GenomicAnnotation > (broad.core.annotation) | EigenvalueDecomposition (Jama) | H | PearsonDistance (broad.pda.geneexpression.clustering) | SplitByName (broad.pda.differentialExpression) |
| AnnotationReaderFactory (broad.core.annotation) | EmpiricalDistribution (broad.core.math) |  | PermutationDistribution (broad.core.math) | SplitFastqByIndex (broad.pda.seq.fastq) |
| AnnotationUtils (umms.core.utils) | EndInWindowCountScore (umms.core.model.score) | HairpinKmer (broad.core.primer3) | PermutationNotFoundException (umms.core.coordinatesystem) | SplitFile (broad.core.siphy.tools.conservation) |
| AnnotationUtils (broad.core.annotation) | EnrichmentMetric (broad.pda.enrichment) | HierarchicalClustering (broad.pda.geneexpression.clustering) | Permutations (broad.pda.differentialExpression) | StartInWindowCountScore (umms.core.model.score) |
| ANOVA (broad.core.math) | EnrichmentUtils (broad.pda.enrichment) | I | PhastconsReader (broad.core.siphy) | StationaryDistributionIO (broad.core.siphy) |
| AverageBranchLength (broad.core.siphy.tools.conservation) | ESAT (umms.esat) |  | PhylipInterleavedMultipleAlignmentIO (broad.core.multiplealignment) | Statistics (broad.core.math) |
| B | ESATDistanceCalculator (umms.esat) | IllegalTreeOperationException (broad.core.datastructures) | PhylipSequencialMultipleAlignmentIO (broad.core.multiplealignment) | StoredAlignmentStats (broad.pda.seq.alignment) |
|  | EstimateOmegaPerExon (broad.core.siphy.tools.conservation) | IncompleteMethodImplementationException (umms.core.exception) | PiHMM (broad.core.siphy) | Annotation.Strand (umms.core.annotation) |
| BadModelException (broad.core.hmm) | EuclideanDistance (broad.pda.geneexpression.clustering) | Interval (broad.core.datastructures) | Pileup (broad.pda.seq.clip) | StringParser (broad.core.parser) |
| BasicAnnotation (umms.core.annotation) | EvolutionaryModel (broad.core.siphy) | IntervalTree< V > (broad.core.datastructures) | PlotAggregateRegions (broad.pda.seq.rap) | SumScore (umms.core.model.score) |
| BasicAnnotationReader (broad.core.annotation) | EvolutionaryModelParameters (broad.core.siphy) | J | PoissonEnrichmentScore (umms.core.model.score) | T |
| BasicGenomicAnnotation (broad.core.annotation) | ExpressionExperimentInfo (broad.pda.geneexpression) |  | PositionWeightColumn (broad.core.motif) |  |
| BasicGenomicAnnotationReader (broad.core.annotation) | ExtractGenes (broad.projection.gsa) | JCSAlignmentModel (umms.core.model) | PositionWeightMatrix (broad.core.motif) | TabbedReader (umms.core.general) |
| BasicLightweightAnnotation (broad.core.annotation) | Extractor (broad.core.sequence) | JCSCache (broad.core.datastructures) | PositionWeightMatrixIO (broad.core.motif) | TracebackNode (jaligner) |
| BasicScanStatisticScore (umms.core.model.score) | ExtractSamplesAndLabel (broad.pda.geneexpression.agilent) | JCSCache.JCSNodeIterator (broad.core.datastructures) | PositionWeightMatrixModel (broad.core.siphy) | TranscribedRegions (broad.core.sequence) |
| BasicTwoSubjectAnnotation (broad.core.annotation) | ExtractSequence (broad.pda.rnai) | K | PrecomputeDataAlignmentStats (broad.pda.seq.segmentation) | TranscribedSequenceComposition (broad.core.sequence) |
| BatchedMultiSampleScanPeakCaller (broad.pda.seq.protection) | F |  | Predicates (umms.core.general) | TranscriptInGenomicSpace (umms.core.coordinatesystem) |
| BatchNorm (broad.pda.differentialExpression) |  | Kmeans (broad.pda.geneexpression.clustering) | PreprocessDataSet (broad.pda.geneexpression) | TranscriptomeCommandLineProgram (broad.pda.seq.rap.rna) |
| BatchSubmitEstimateExon (broad.core.siphy.tools.conservation) | TabbedReader.Factory< T > (umms.core.general) | KSTest (broad.core.math) | Primer3Exception (broad.core.primer3) | TranscriptomeScoringProgram (broad.pda.seq.rap.rna) |
| BED (broad.core.annotation) | FASTA (jaligner.formats) | L | Primer3PickingSummary (broad.core.primer3) | TranscriptomeSpace (umms.core.coordinatesystem) |
| BEDFileParser (broad.pda.annotation) | FastaExtendPeaks (broad.pda.seq.clip) |  | Primer3PrimerPickingResult (broad.core.primer3) | TreeScaler (broad.core.siphy) |
| BEDGraph (broad.core.annotation) | FastaHandler (broad.core.sequence) | LengthScore (umms.core.model.score) | PrimerUtils (broad.core.primer3) | TreeScalerIO (broad.core.siphy) |
| BEDGraphReader (broad.core.annotation) | FastaMultipleAlignmentIO (broad.core.multiplealignment) | LightweightGenomicAnnotation (broad.core.annotation) | ProcessDataset (broad.pda.geneexpression.agilent.analysis) | TrimFastqReads (broad.pda.samtools) |
| BEDReader (broad.core.annotation) | FastaParser (broad.core.sequence) | LocationAwareMatrix (broad.pda.datastructures) | PSLRecord (umms.core.annotation) | TwoSampleScanSkellamPeakCaller (broad.pda.seq.protection) |
| BinomialEnrichmentScore (umms.core.model.score) | FastaQualityBinaryIO (broad.core.sequence) | Locus (broad.pda.annotation) | PSLXToSAM (broad.pda.tools) | TwoSubjectAnnotation (broad.core.annotation) |
| BuildRatioNullDistribution (broad.pda.seq.rap) | FastaQualityHandler (broad.core.sequence) | Loess (broad.core.math) | PWMUtils (broad.core.motif) | U |
| BuildScriptureCoordinateSpace (umms.core.scripture) | FastaQualityIndex (broad.core.sequence) | LUDecomposition (Jama) | Q |  |
| C | FastaQualityParser (broad.core.sequence) | M |  | UnableToFitException (broad.core.siphy) |
|  | FastaSequenceIO (broad.core.sequence) |  | QCAgilentResults (broad.pda.geneexpression.agilent) | UnfairDie (broad.core.math) |
| CalculateCoverageStats (broad.pda.seq.rap) | FastqLibraryStats (broad.pda.countreads) | MAFAlignment (broad.core.multiplealignment) | QRDecomposition (Jama) | UniqueMappedReadsFilter (umms.core.readFilters) |
| Cell (jaligner) | FastqParser (broad.pda.seq.fastq) | MAFIO (broad.core.multiplealignment) | R | UnmarkDuplicates (broad.pda.seq.rap) |
| CholeskyDecomposition (Jama) | FastqSequence (broad.pda.seq.fastq) | MakeCLSFiles (broad.pda.geneexpression.agilent) |  | FeatureSequenceAlignment.UnorderedGenePair (umms.core.alignment) |
| Chromosome (broad.pda.feature.genome) | FastqSplitterByBarcode (broad.pda.seq.fastq) | MakeCLSFromDescription (broad.pda.geneexpression.agilent) | RankOrder (broad.pda.geneexpression.clustering) | W |
| ChromosomeInconsistencyException (umms.core.alignment) | FDRDistribution (broad.core.math) | MakePositionalGeneSets (broad.pda.differentialExpression) | RAPPeakCaller (broad.pda.seq.rap) |  |
| ChromosomeStringComparator (broad.core.util) | Feature (broad.core.annotation) | MannWhitney (broad.core.math) | RatioPermutationPeakCaller (broad.pda.seq.rap) | Wilcox2Sample (broad.core.math) |
| ChromosomeWithBubblesJGraphT (broad.pda.seq.graph) | FeatureSequenceAlignment (umms.core.alignment) | MannWhitneyPermutations (broad.core.math) | RatioScore (umms.core.model.score) | Window (umms.core.feature) |
| CisVsTrans (broad.pda.differentialExpression) | AlignmentModel.FilteredIterator (umms.core.model) | MappingQualityFilter (umms.core.readFilters) | ReadCountNormalizer (broad.pda.seq.segmentation) | WindowAllScore (umms.core.model.score) |
| ClassifyAgilentLincProbes (broad.pda.geneexpression.agilent) | JCSAlignmentModel.FilteredIterator (umms.core.model) | MarkovModel< T > (broad.core.hmm) | ReadEndsInFilter (umms.core.readFilters) | WindowProcessor< S extends WindowScore > (umms.core.model.score) |
| CloseableFilterIterator< T > (umms.core.general) | FilterFastqReads (broad.pda.samtools) | MarkovState< T > (broad.core.hmm) | ReadFilter< T > (broad.pda.seq.segmentation) | WindowScore (umms.core.model.score) |
| CLUSTAL (jaligner.formats) | FilterGCT (broad.pda.geneexpression) | Markups (jaligner) | ReadSimulator (broad.pda.seq.protection) | WindowScoreIterator< T extends WindowScore > (umms.core.model.score) |
| Cluster (broad.pda.geneexpression.clustering) | FilterGCTFile (broad.pda.geneexpression) | MaskedGenomicSpace (umms.core.coordinatesystem) | ReadSimulator2 (broad.pda.seq.protection) | WindowScores (broad.pda.seq.protection) |
| ClusterDistanceFunction (broad.pda.geneexpression.clustering) | FilterMatrixByMinCutoff (broad.pda.geneexpression.agilent) | Maths (Jama.util) | ReadStartsInFilter (umms.core.readFilters) | WindowSlider (broad.core.sequence) |
| CLUtil (broad.core.util) | FilterPairedReadsByMAQ (broad.pda.samtools) | MathUtil (broad.core.math) | ReadsToReconstructFilter (nextgen.core.readFilters) | JCSAlignmentModel.WrapAlignmentCountIterator (umms.core.model) |
| CollapseByIntersection (broad.core.util) | FindDiffrentialEnrichedRegions (broad.pda.seq.chip) | MatricesCompartor (jaligner.matrix) | ReconstructionComparer (broad.pda.seq.segmentation.utils) | AlignmentModel.WrapAlignmentCountIterator (umms.core.model) |
| CollapsedGeneLengths (umms.esat) | FindEnrichedDepletedRegions (broad.pda.rap.misc) | Matrix (jaligner.matrix) | RepeatMaskerAnnotation (broad.core.annotation) | WrappedIGVIterator (broad.pda.seq.segmentation) |
| CollapseProbesByMinPValue (broad.pda.geneexpression.agilent) | FindProbesForGeneSet (broad.pda.geneexpression.agilent) | Matrix (Jama) | RepeatMaskerAnnotationReader (broad.core.annotation) | WrappedIterator (broad.pda.samtools) |
| CollapseProjections (broad.pda.differentialExpression) | FisherCombinedProbabilityTest (broad.core.math) | MatrixGenerator (jaligner.matrix) | RestrictionEnzyme (broad.core.primer3) | SingleEndAlignment.WrapSamRecord (umms.core.alignment) |
| CollectAnnotationEnrichments (broad.pda.seq.rap) | Fit (broad.core.siphy) | MatrixLoaderException (jaligner.matrix) | ReversibleIterator< E > (broad.core.datastructures) | f |
| CollectRnaSeqMetrics (broad.pda.seq.rap.rna) | FixedControlDifferentialExpression (broad.pda.differentialExpression) | MatrixWithHeaders (broad.core.datastructures) | IntervalTree< V >.RevIterator (broad.core.datastructures) |  |
| CombinationGenerator (broad.core.math) | FoldSignificance (broad.pda.differentialExpression) | MatrixWithHeaderUtils (broad.core.datastructures) | RNAiGeneAnnotation (broad.pda.rnai) | functionalAnnotation (broad.pda.gene) |
| CommandLineParser (broad.core.parser) | Format (jaligner.formats) | MaximumContiguousSubsequence (broad.core.annotation) | RunAllGSEABatch (broad.pda.differentialExpression) | g |
| Commons (jaligner.util) | FormatFactory (jaligner.formats) | MemeMotif (broad.core.motif.meme) | RunDifferentialExpression (broad.pda.differentialExpression) |  |
| CompoundInterval (umms.core.annotation) | FragmentAlignment (umms.core.alignment) | MemeResultReader (broad.core.motif.meme) | RunGSEA (broad.pda.differentialExpression) | geneExpUtils (broad.pda.geneexpression) |
| ComputeConservedMotifs (broad.core.motif.misc) | FullyContainedFilter (umms.core.annotation.filter) | MergeGSEAOutputIntoMatrix (broad.pda.differentialExpression) | RuntimeIOException (umms.core.exception) | i |
| ComputeCorrelationOfReplicates (broad.pda.geneexpression.clustering) | IntervalTree< V >.FwdIterator (broad.core.datastructures) | MultipleAlignment (broad.core.multiplealignment) | RunTimeSeries (broad.pda.differentialExpression) |  |
| ComputeEnrichments (broad.pda.seq.chip) | FWERDistribution (broad.pda.differentialExpression) | MultipleAlignmentFactory (broad.core.multiplealignment) | S | isPCRLike (broad.core.primer3) |
|  |  |  |  |  |
|  |  |  | SameOrientationFilter (umms.core.readFilters) |  |
|  |  |  |  |  |

A | B | C | D | E | F | G | H | I | J | K | L | M | N | O | P | Q | R | S | T | U | W


---

Generated on Fri Aug 15 2014 15:11:16 for ESAT by  

 1.8.7
